# Motherhood after cancer: fertility and utilisation of fertility-preservation methods

**DOI:** 10.1007/s00404-020-05563-w

**Published:** 2020-05-06

**Authors:** Maren Goeckenjan, A. Freis, K. Glaß, J. Schaar, I. Trinkaus, S. Torka, P. Wimberger, A. Germeyer

**Affiliations:** 1 Department for Gynaecology and Obstetrics, Technische Universität Dresden, University Hospital, Dresden, Germany; 2 Department for Gynaecological Endocrinology and Reproductive Medicine, University Hospital of Heidelberg, Heidelberg, Germany

**Keywords:** Fertility preservation, Cancer, Ovarian cryopreservation, GnRH agonists, Follow-up

## Abstract

**Purpose:**

Due to modern and individualised treatments, women at reproductive age have a high survival rate after cancer therapy. What are pregnancy and birth rates of women after cancer and how often do they use cryopreserved ovarian tissue or gametes?

**Methods:**

From 2007 to 2015, 162 women aged 26.7 ± 6.9 years were counselled for fertility preservation at a single University Fertility Centre. A questionnaire study was performed in average 3 and 6 years after the diagnosis of cancer. The women were asked about their fertility, partnership, family planning, and pregnancy history. 72 women (51%) answered a written questionnaire in 2016. 59 women were reached again by phone in 2019 (82%).

**Results:**

The preferred method of fertility preservation was ovarian tissue cryopreservation (*n* = 36, 50%); none of the women had ovarian hyperstimulation in order to cryopreserve oocytes. About 3 years after treatment, 37 women of 72 women (51%) of the women with a mean age of 29.9 years had a strong wish to conceive. 21/72 (29%) had actively tried to conceive after successful cancer treatment; eight women (11%) were already pregnant or had children. Six years after cancer diagnosis 16/59 (27%) women had ongoing anticancer treatment. 12/59 (20%) were pregnant or had children, while 39% (23/59) had no menstrual cycle. Only one woman used her cryopreserved ovarian tissue, but did not become pregnant.

**Conclusion:**

After cancer and gonadotoxic treatment, women’s desire to have a child is substantial. In this study, the rate of spontaneous pregnancies and births was 20% 6 years after gonadotoxic therapies. Not every woman, however, has the opportunity to conceive: factors impairing fertility include ongoing cancer treatment or persistent disease, no partner, no menstrual cycle, as well as other reasons for infertility.

## Introduction

Today’s procedures for counselling women with newly diagnosed cancer for fertility preservation are fairly standardised. Due to high survival rates and high quality of life after cancer, women should be given the opportunity to plan motherhood after cancer actively. Methods to preserve fertility in spite of gonadotoxic treatments are well established and available nationwide in Germany. Since the foundation of a network on fertility preservation in German-speaking countries, *Ferti*PROTEKT e.V. in 2006, more than 130 centres are counselling women with planned gonadotoxic therapies in cooperation with oncologic medical centres. The network defines objectives for counselling and options for fertility preservation: Patients of reproductive age should be advised about their potential decline in fertility due to the gonadotoxic treatment before its initiation. Effective fertility preservation methods should be discussed and offered [1]. In spite of these efforts to standardise the procedures, the use of fertility-preservation methods differs from centre to centre and regionally.

The registered data of the participating centres show that through 2013, more than 5000 women were counselled and more than 4000 received fertility protective treatments [2]. These treatments include medical therapy with ovarian suppression via gonadotropin-releasing hormone (GnRH) agonists, cryopreservation of oocytes after controlled ovarian hyperstimulation, and cryopreservation of ovarian tissue. In many cases, more than one treatment for fertility preservation was used.

Since 2017, German national guidelines with standardised recommendations have been available [3]. International practical recommendations have also been published [4]. Table 1 outlines the currently recommended treatment procedures for women of reproductive age with the most frequent cancer diagnoses. At the time of the study, the methods of fertility preservation were not covered by the national health insurance companies. The costs for controlled ovarian hyperstimulation and aspiration of oocytes as well as cryopreservation of unfertilized or fertilised oocytes for some years were calculated with approximately 1500 € for medication, 1500 € for aspiration of oocytes, and storage of oocytes (300–400 € per year) [1]. The costs for ovarian tissue cryopreservation were calculated with approximately 1000 €for laparoscopic surgery, and shipping, tissue preparation, and storage with 300–400 € per year.

**Table 1:** Recommended fertility preservation methods for women with planned gonadotoxic treatment (German guidelines for fertility preservation [3], Schüring et al. 2018 [4])

Indication	Treatment		Comment
Hodgkin lymphoma	Fertility preservation is recommended in women with high risk of premature ovarian insufficiency (POI)		The time frame between diagnosis and treatment is often short. Controlled ovarian stimulation and cryopreservation of oocytes is possible, if treatment can be delayed by 2–3 weeks for ovarian stimulation
	Fertility preservation may be considered in women with low or moderate risk of POI		
	GnRH agonists, cryopreservation of ovarian tissue and oocytes after ovarian stimulation are adequate options		
	A combination of fertility preserving methods is possible		
Breast cancer	Fertility preservation is recommended		The individual impact on fertility is dependent on the complex oncologic treatment: chemotherapy, antihormonal treatment, time interval until pregnancy, and ovarian aging
	GnRH agonists, cryopreservation of ovarian tissue and oocytes after ovarian stimulation are adequate options		
	A combination of fertility preserving methods is possible		
Autoimmune diseases	Fertility preservation is recommended before cyclophosphamide treatment		Treatment with GnRH agonists is now established without a specialised counselling process. Due to the need for urgent treatment, methods with cryopreservation are often not available
	GnRH agonists are a possible option		
	Cryopreservation of ovarian tissue and oocytes can be applied in individual cases		
Non-Hodgkin lymphoma/leukaemia	Fertility preservation is recommended depending on prognosis and treatment		Cryopreservation of ovarian tissue and oocytes after ovarian stimulation are not recommended because of the risk of ovarian metastasis
	GnRH agonists are an option		
Ewing sarcoma	Fertility preservation is recommended depending on the clinical situation		The risk of ovarian metastasis must be discussed
	GnRH agonists and cryopreservation of oocytes can be considered		
	Cryopreservation of ovarian tissue is possible		

Throughout the individual decision-making progress, each woman is given information about the prognosis of her disease, the planned treatment, and its impact on fertility. If a high risk for infertility is suspected and/or the woman has a strong wish to bear her own child, fertility-preservation methods are recommended. The final decision is highly individual and made after an intensive counselling including ethical implications [5]. The interdisciplinary coordination of fertility-preservation methods and oncological treatment, the confrontation of the patient with a possibly life-threatening diagnosis, and the necessity of reaching a decision in a short time create a special burden for the counselling. In addition to these aspects and the financial burden, medical risks must be calculated against the possible benefits of the treatment.

At the University Fertility Centre in Dresden, the counselling of female cancer patients and women with autoimmune disease prior to gonadotoxic treatments has been documented in a local registry since 2007. Recommended fertility preservation options included all established treatments, including GnRH agonists, ovarian cryopreservation, and cryopreservation of oocytes. The low utilisation rate of hormonal stimulation and cryopreservation of oocytes in the Fertility Centre in Dresden at the study period was due to the high costs of this method and the longer time required compared to ovarian tissue cryopreservation. This may change in the future as the German government recently proclaimed that the costs of cryopreservation of gametes will be covered by the national health insurance. The first birth of a child conceived after autotransplantation of cryopreserved ovarian tissue took place in Dresden [6]. As this novel option for fertility preservation was elaborately discussed and presented in the regional media, cryopreservation of ovarian tissue was the preferred fertility preservation method to be chosen in the counselling process at the University Fertility Centre in Dresden at the time of the study. Current data after autotransplantation of ovarian tissue show that the ovaries resume function in about 80–85% of women and that pregnancies can be achieved spontaneously [7]. The cryopreservations and autotransplantations were performed in cooperation with the University Hospital of Erlangen.

Follow-up data after using fertility-preservation methods are still sparse. The first major study of long-term follow-up after fertility preservation was published only recently [8]. The data presented in this article show a follow-up after fertility preservation counselling in a single fertility centre in Germany.

## Material and methods

The data of 162 women counselled for fertility preservation between January 2007 and December 2015 at the University Fertility Centre in Dresden were collected in a registry. In 2016, a prospective questionnaire follow-up study was initiated in cooperation with the University Hospital of Heidelberg. The written questionnaire was developed by A. Germeyer and is not validated up to date.

The written questionnaire has 37 items, which include the following topics:

- eight questions: person and disease;
- six questions: family, partnership, and family planning;
- six questions: biological fertility, menstrual cycle, contraception, and hormonal replacement therapy;
- nine questions: reproduction, fertility, and infertility;
- four questions: counselling for fertility preservation; and
- four questions: pregnancies before and after the diagnosis of cancer.

**Fig. 1:**
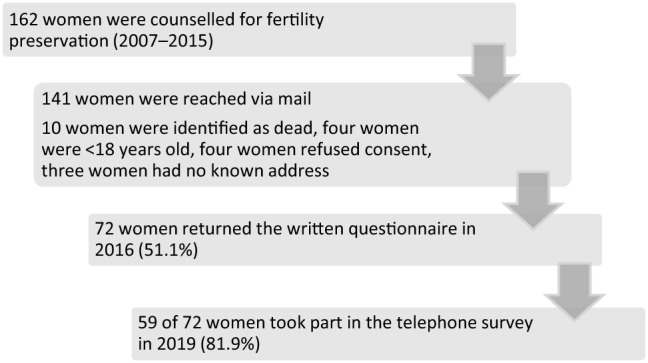
Flowchart of study participants

The study protocol was approved by the local ethics committee (EK 314072015). Inclusion criteria were: documented counselling for fertility preservation, age > 18 years, and written informed consent to the study protocol. The first follow-up was performed via postal mailing. The return rate of the paper questionnaire was 51% in 2016. The study-inclusion flowchart of the study is shown in Fig. 1. A second follow-up survey was conducted by telephone in 2019; at that time, 59 of 72 women were reached again (82%). Seven questions regarding the patients’ general health, possible ongoing cancer treatment, current or past pregnancies and births, since fertility preservation counselling, utilisation of cryopreserved material, partnership, and infertility were asked by two medical doctors in a standardised verbal interview.

The characteristics of the women counselled and treated with the methods of fertility preservation in the fertility centre are shown in Table 2. This table indicates that the study group does not differ statistically from all women who were counselled for fertility preservation in the *Ferti*PROTEKT network e.V.

**Table 2:** Characterisation of study group (*n* = 72) compared to group of counselled women for fertility preservation (*n* = 162) at the time of the questionnaire mailing and rounded data of the FertiPROTEKT network from 2007–2015 [1]

	All women counselled for fertility preservation 2007–2015	Study group of women (answered questionnaire) in 2016	Statistical analysis (*p* value)	Data of the ‘*Ferti*PROTEKT’ network 2007–2015
Number of women	162	72	–	≈ 7150
Mean age in years at counselling /diagnosis of cancer	26.7 ± 6.9 (6–40)	27.1 ± 6.3 (15–39)	0.689	28
Mean age in years at questionnaire study (8/2016)	30.6 ± 7.2 (8–48)	30.4 ± 6.4 (17–44)	0.807	–
Time interval between counselling and time of contact (sent and/or answered questionnaire)	3.8 ± 2.4 (1–9)	3.1 ± 2.2 (1–9)	0.064	–

	In % (*n* =)	In % (*n* =)	Statistical analysis (*p* value)	In %
Most frequent diagnoses				
Breast cancer	35.2 (57)	37.5 (27)	0.735	37
Hodgkin lymphoma	26.5 (43)	36.1 (26)	0.154	26 (incl. NHL)
Autoimmune diseases	9.3 (15)	1.4 (1)	0.004	7
Non-Hodgkin lymphoma	7.4 (12)	8.3 (6)	0.807	Not determined
Leukaemia	5.6 (9)	6.9 (5)	0.681	5
Ewing sarcoma	3.1 (5)	4.2 (3)	0.676	Not determined
Fertility preservation methods^a^				
GnRH	58.0 (94)	62.5 (45)	0.552	47
Cryopreservation of ovarian tissue	40.1 (65)	50.0 (36)	0.161	33
Cryopreservation of oocytes	1.9 (3)	0	0.083	17
Transposition of ovaries	0.6 (1)	1.4 (1)	0.556	2

^a^Multiple treatment possible, most often GnRH agonists in combination with cryopreservation of ovarian tissue

Differences in study groups were compared using Student’s *t* tests and Mann–Whitney *U* test as appropriate. *p* < 0.05 was considered statistically significant. SPSS-V 25.0 was used.

## Results

### Results of the written questionnaire, on average 3.1 years after diagnosis

Of the 162 patients who were counselled for fertility preservation in 2007–2015, ten women were known to have died within 3 years (6.2%). 141 patients could be contacted by mail, and 72 answered the questionnaire and sent it back, resulting in a response rate of more than 51%. The median latency between the diagnosis of cancer and the questionnaire study was 3.1 ± 2.2 years.

The study group generally did not differ from the group of all counselled patients in the same fertility centre within that time frame (Table 2). The data were also compared to the group of women counselled and documented by the *Ferti*PROTEKT network e.V. [1]. Only the group of women with autoimmune diseases was significantly smaller in the study group than the group of all women counselled. This difference may be explained by the special situation of women with benign diseases. These patients usually decide to use ovarian downregulation with GnRH agonist rather than utilising cryopreservation methods.

**Fig. 2:**
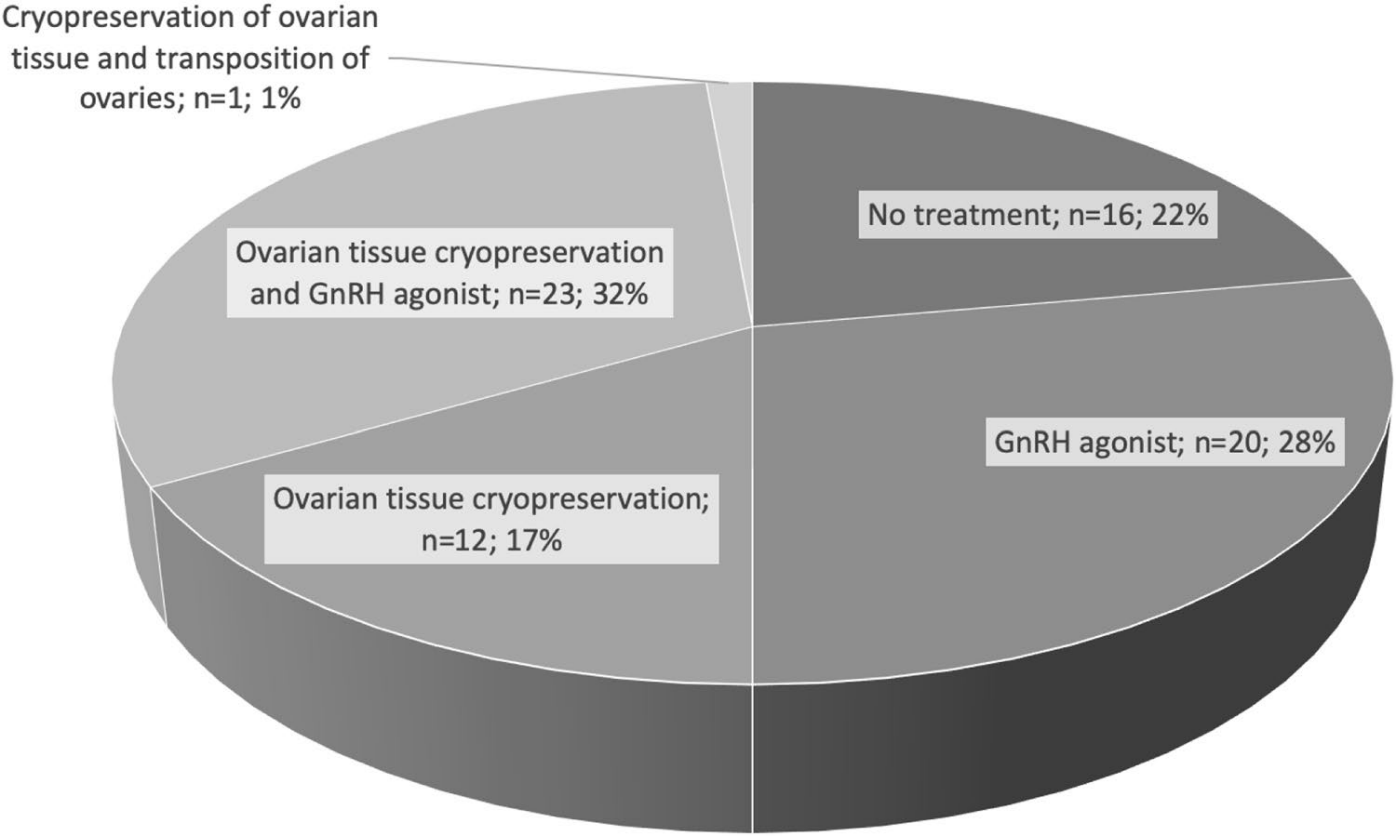
Fertility preservation decisions by number and percentage of women in the questionnaire study (*n* = 72)

Figure 2 shows the fertility preservation treatment utilised by the women in the questionnaire study. 22.2% of the women (16/72) had decided not to have any fertility preservation treatment, while every second woman underwent ovarian cryopreservation (36/72). Almost every third woman in the study group (23/72) combined the medical treatment of a GnRH agonist with the cryopreservation of ovarian tissue.

As part of the questionnaire, women were asked about their general wish to have a child. Table 3 shows the results of the entire study group and subgroups. Women who decided to have ovarian cryopreservation declared 3 years later a stronger wish for their own child; however, this difference was not significant (*p* = 0.101).

**Table 3:** Intensity of the desire to have a child three years after counselling in the study group, as well as in the subgroups of women, who underwent invasive fertility preservation versus women, who only used GnRH agonist or no treatment: statistical analysis of differences between the two subgroups (*n* = 72)

Intensity of desire to have a child	All women (*n* = 72)	Women after cryopreservation of the ovary (*n* = 36)	Women with GnRH agonists or no treatment for fertility preservation (*n* = 36)	*p* value (Mann-Whitney *U *test)
None	8 (11.1%)	2 (5.6%)	6 (16.7%)	0.136 (n.s.)
Weak	17 (23.6%)	7 (19.4%)	10 (27.8%)	0.408 (n.s.)
Median	8 (11.1%)	5 (13.9%)	3 (8.3%)	0.456 (n.s.)
Strong	37 (51.4%)	22 (61.1%)	15 (41.7%)	0.101 (n.s.)
No response	2 (2.8%)	0	2 (5.6%)	0.154 (n.s.)

Twenty-one women had already tried to conceive (29%). Of these, eight women became pregnant spontaneously, while 13 women stated that in spite of unprotected sexual intercourse, no pregnancy occurred.

57/72 women were in a stable partnership (79%); 12 of these women (17%) had a new partnership after cancer. 55/72 women had had no examination of their fertility status after cancer treatment at the time of answering the questionnaire. 7/15 women with fertility check-ups had been given a diagnosis of infertility after gynaecological and/or andrological examinations (47%). Two women had already undergone infertility treatment with artificial reproductive technologies, but no pregnancy occurred. Two women did not respond to the questions about their fertility status.

Four women reported having had a total of five children after their diagnosis of cancer; in addition, four women were pregnant at the time of the questionnaire. The data of the 8/72 women are depicted in Table 4. All women who were pregnant or already had a child were at least 3 years post-cancer diagnosis (6 years on average). The age at diagnosis of cancer ranged from 20 to 29 years. Six of the eight women had ovarian tissue cryopreserved, but had not used it for autotransplantation. All pregnancies occurred spontaneously without infertility treatment or use of cryopreserved tissue.

**Table 4:** Clinical data of the eight women with pregnancies until their responses to the written questionnaire (median age at pregnancy 29 years)

	Pregnancy	Cancer, anticancer therapy	Cryo-preservation	GnRH agonists	Age at diagnosis (age at pregnancy) in years	Interval to pregnancy (years since diagnosis)	Comments
1 BK	Birth of a healthy child	Breast cancer (Triple negative pT2, pN1a [1/11 LK], L0, V0, G3) 4 × EC, Docetaxel	+	−	23 (27)	4	No utilisation of tissue
2 SC	Birth of two healthy children	Hodgkin lymphoma Stage IIIB, 8 × BEACOPP esc	+	+	21 (28)	7	No utilisation of tissue
3 BA	Birth of a healthy child	NHL CHOP, Rituximab	−	+	28 (36)	8	–
4 BM	Birth of a healthy child	Hodgkin lymphoma stage IB, 2 ABVD	−	+	24 (30)	6	–
5 PL	Pregnant	Hodgkin lymphoma stage 2A, 4 × ABVD	+	+	20 (27)	7	No utilisation of tissue
6 BB	Pregnant	Osteosarcoma, 7 × VIDE	+	+	21 (27)	6	No utilisation of tissue
7 FK	Pregnant	Hodgkin lymphoma stage III, 6 × BEACOPP	+	−	29 (32)	3	No utilisation of tissue
8 GU	Pregnant	Breast cancer (pT1c, pN0, pM0, V0, L0, G3, E and P positive, Her2neu neg., 4 × EC, Taxotere)	+	+	27 (34)	7	No utilisation of tissue

*EC* Epirubicin Cyclophosphamid; *BEACOPP esc* Bleomycin, Etoposid, Adriamycin, Cyclophosphamide, Oncovin, Procarbazine, Prednisone in dose-escalation; *NHL* nonHodgkin-Lymphoma; *CHOP* Cyclophosphamide, Hydroxydaunorubicin, Oncovin, Prednisone; *ABVD* Adriamycine, Bleomycin, Vinblastine, Dacarbazine; *VIDE* Vincristine, Ifosfamide, Doxorubicin, Etoposide

All women without a wish to conceive were explicitly asked in the questionnaire to explain possible influences on their family planning. Their reasons for not wishing to conceive at the time of the questionnaire were as follows: no partnership (*n* = 7), homosexual partnership and no existing sperm donor (*n* = 2), fear of relapse of the disease (*n* = 5), and fear of having a child with impairments (*n* = 6). Eight women stated that they no longer wanted to conceive (11%); of these, four women already had children and had completed their family planning. One woman had nine miscarriages and no live birth.

**Fig. 3:**
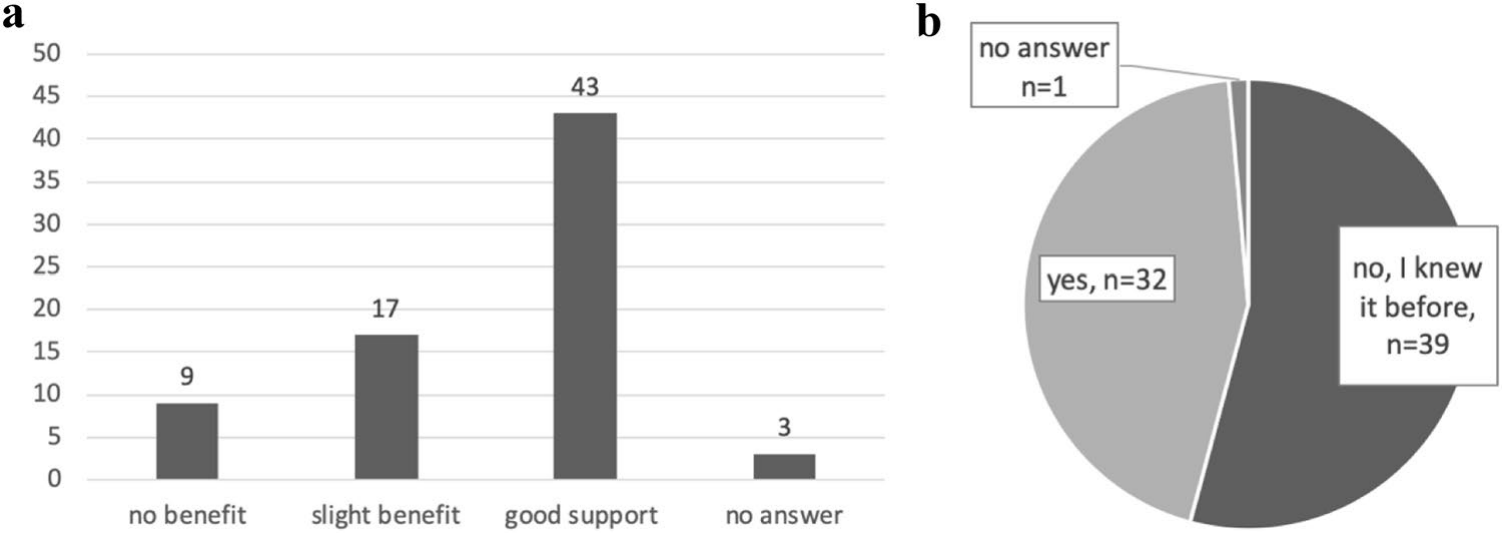
**a** Do you feel that the counselling for fertility preservation was helpfull at the time of diagnosis? Number of answers (*n* = 72). **b** Was it the first time you learned about the side effect of infertility due to anticancer treatment during the fertility counselling? Number of answers (*n* = 72)

The women were asked about their opinions on the counselling for fertility preservation at the time of diagnosis (Fig. 3a, b). Eighty-six percent of them would recommend the counselling to other women in similar situations.

### Second follow-up interview by telephone, on average 6.0 years after diagnosis

In April 2019, 59/72 women were reached by telephone for a follow-up interview. The mean age of the women at this time was 32.9 ± 6.5 (21–46) years. Of these, 17 women did not use a fertility preservation method at the time of first counselling (28.8%). *N* = 33/59 (56.0%) had undergone ovarian cryopreservation and *N* = 33/59 (56.0%) had used the medical treatment with GnRH agonists; 19/59 (32.2%) used both methods of fertility preservation.

**Fig. 4:**
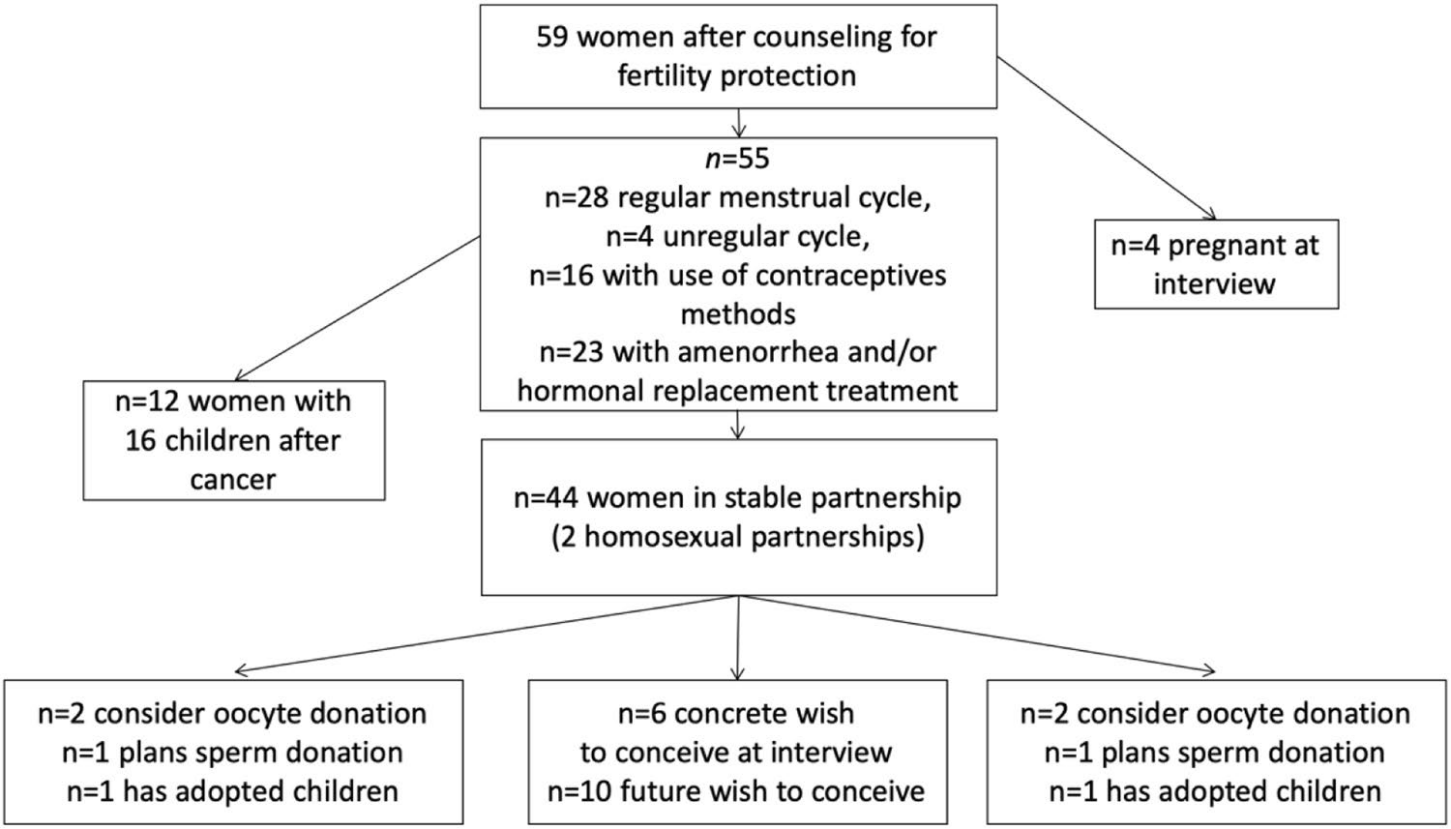
Flowchart showing fertility history 6 years after diagnosis of cancer (*n* = 59)

The fertility history of the women is shown in Fig. 4. In the interview, the women were explicitly asked about an ongoing or recurrent oncological treatment; 16/59 (27%) had anticancer treatment and, for medical reasons, were not permitted to become pregnant. 23/59 women (39%) said that they had amenorrhea or hormonal replacement therapy after premature ovarian insufficiency. Of 55 women who were not pregnant at the time of the interview, 28 described their cycle as regular and four as irregular. Sixteen of the women used contraceptive methods.

53/59 women rated their overall health as ‘good’ or ‘very good’ (90%) even with ongoing treatment. Twelve women had given birth to 16 healthy children until the time of the second counselling, and four women were pregnant at the time of the interview. None of the pregnant women had used their cryopreserved ovarian tissue. All pregnancies occurred spontaneously (*n* = 12, 2%). Six women said that they were planning to become pregnant (10%).

In the telephone interview, the patients expressed conflicting interests concerning their family planning. On one side, women described personal and medical situations which make pregnancies possible: ‘I am in very good health’ (*n* = 46), ‘I live in a stable partnership’ (*n* = 44), and ‘I have a regular menstrual cycle’ (*n* = 28).

On the other hand, aspects which do not allow a pregnancy were clearly stated: ‘The disease makes a pregnancy impossible’ (*n* = 16), ‘No pregnancy because no stable partnership’ (*n* = 15), ‘I have no ovulation anymore’ (*n* = 23), and ‘I use contraceptive methods’ (*n* = 16).

Of the 33 women, who had ovarian tissue stored at the time of the interviewed 6 years after diagnosis, five (15%) had already decided to dispose of their tissue. Three women chose to dispose of the ovarian tissue, because they had already completed their family planning, one woman because of her age (44 years), and one woman with bone metastasis because of her prognosis. One woman aged 39 years used the ovarian tissue and had two trials of intracytoplasmic sperm injection because of infertility of her partner; however, no pregnancy occurred. 27 of 33 women with a mean age of 30.3 years still had their ovarian tissue cryopreserved; of these, six women had active cancer disease, and six stated that their family planning had been completed. Sixteen women with stored ovarian tissue planned to conceive in the future.

Women expressed emotional reasons to further preserve the stored ovarian tissue in the telephone interviews. Examples are: one 35-year-old woman with liver and brain metastases after primary diagnosis of breast cancer said: ‘Still, I really want to keep the tissue cryopreserved—I cannot throw away a part of my body.’ Other women claimed the following reasons for ongoing cryopreservation: ‘The knowledge that there is still some ovarian tissue makes a big difference for me’, ‘The tissue gives me a reassuring positive feeling’, ‘It is important for my psychological stability’, and ‘I like the thought that I could use the tissue for hormonal treatment—if necessary’.

## Discussion

Many women today have the opportunity to plan a family after surviving their cancer, as modern individual anticancer treatments have led to high survival rates in young cancer patients. Counselling regarding fertility preservation techniques at the time of diagnosis before initiating gonadotoxic treatment became a standard procedure. Nevertheless, follow-up data after counselling for fertility preservation are still sparse.

We present a follow-up study after fertility preservation counselling in a single university fertility centre. Fifty percent of the women who participated in the questionnaire study had ovarian tissue cryopreserved after the fertility preservation counselling. None of the women, which answered the questionnaire, had performed ovarian stimulation for cryopreservation of oocytes or embryos in our centre.

Although our data show that a high percentage of women with spontaneous pregnancies used GnRH agonists, this cannot be interpreted as an effect of ovarian suppression at the time of chemotherapy. The debate about the effect of GnRH agonists in protecting ovarian function against gonadotoxic treatment is still ongoing [9], and further prospective data are needed. Still, the data presented in this small study are reassuring. Even after the medical recommendation and individual decision to use fertility protection methods, spontaneous pregnancies can occur in a high percentage of women with unprotected sexual intercourse.

A current study from Sweden describes the long-term follow-up of 1254 girls and women after fertility preservation counselling in the period between 1998 and 2018 [8]. Women with benign and malignant diseases were counselled. In this study, the majority of women chose the cryopreservation of oocytes as their method of fertility preservation (*n* = 538, 73%). This differs to the setting in the University Fertility Centre in Dresden, where hormonal stimulation and cryopreservation of oocytes or embryos were used only rarely, as the estimated costs for this treatment are about 3000–4000 Euros. These costs were required to be fully paid by the patients as the national health insurance did not cover the treatment during the study period. The latest political decision in Germany regarding the national health insurance to cover cryopreservation of gametes and ovarian tissue for fertility preservation reasons is likely to change the decision-making process.

The study by Rodriguez-Wallberg [8] showed that 27% (*n* = 255) of treated women with cancer returned to the fertility centre in Sweden for further diagnostic or therapeutic procedures. Twenty-six women conceived after using cryopreserved ovarian tissue or oocytes. The follow-up time of this study of about 3 years, as well as the percentage of breast cancer, corresponds to our data. The utilisation rates of cryopreserved embryos or oocytes of 8–29% reported by Rodriguez-Wallberg [8] correspond to another European study, in which an utilisation rate of 24% is reported [10]. Interestingly, in this study with 137 women, who underwent hormonal stimulation for fertility preservation reasons between 2003 and 2016, a disposal rate of 12% without utilisation was described for cryopreserved embryos and oocytes in cancer patients. These data resemble the 15% disposal rate of ovarian tissue after 6 years of follow-up in our study (5/33 women). The main clinical difference between using oocytes or embryos and ovarian tissue is the severity of the intervention needed to conceive: In the former, a simple vaginal embryo transfer without risk of transplantation of tumour cells is needed, while the latter requires the laparoscopy and transplantation of autologous ovarian tissue with the theoretical risk of reintroducing tumour cells. Although the medical risks of surgery for removing and transplanting ovarian tissue by laparoscopy are considered low [11], the active decision for the right time to use the tissue and to plan the necessary surgery may be a barrier.

A prospective multicentre study with a cohort of almost 300 women with breast cancer and fertility-preservation methods has already published the baseline data, but not ongoing data of the reported usage of fertility-preservation methods [12]. In a study from Israel, 18/338 women used ovarian tissue for autotransplantation (5.3%) after 6 years [13]. However, in the same study, a 30% delivery rate was reported in 203 women without autotransplantation despite the fact that these women had ovarian tissue cryopreserved. These results correspond to our study: 9/32 women (28.1%) became pregnant spontaneously without autotransplantation, even though they had tissue cryopreserved earlier. The cryopreserved ovarian tissue was used for autotransplantation in only 1 case out of 33 (3.0%), without achieving a pregnancy.

Nevertheless, low utilisation rates of sperm frozen for fertility preservation in men with cancer have also been reported. In a retrospective study, the utilisation rate of cryopreserved sperm was analysed [14]. In a Scottish University Fertility Centre, 264 men with cancer banked sperm prior to chemotherapy or surgery between 2000 and 2017. After a follow-up of 4 years, only 5% of treated men returned to the centre to use their sperm for fertility treatment.

On average 6 years after being diagnosed with cancer, 16/33 women still wish to use the ovarian tissue later. The positive psychological aspects of the cryopreserved ovarian tissue as a fertility reserve were clearly stated by the women. The follow-up period of a mean duration of 6 years in our study is, therefore, still not sufficient to describe the full effects of fertility preservation in young women.

Cryopreserved tissue, oocytes, or embryos make it possible to delay the chance of having children after cancer for several years (‘frozen hope’). On one hand, our study showed a relatively high spontaneous pregnancy rate after cancer: 27.1% of women became pregnant spontaneously (16/59). On the other hand, however, the data indicate that the high rate of persistent amenorrhea of 39% 6 years after treatment is a severe threat to fertility. At the time of the second follow-up, the women were on average 33 years old. These data must be interpreted carefully, as no endocrine follow-up was used to confirm this self-reported diagnosis.

A limitation of our study is the low response rate after 3 years of 51% in the written questionnaire study. Only women who decided to participate in the study could be followed for another 3 years for the second follow-up. This response rate can be explained through the exceptional and emotional situation of counselling on fertility preservation before gonadotoxic treatment. We do not have reliable data about the women who did not participate in our questionnaire study; a response bias by selective participation to the study cannot be ruled out.

**Fig. 5:**
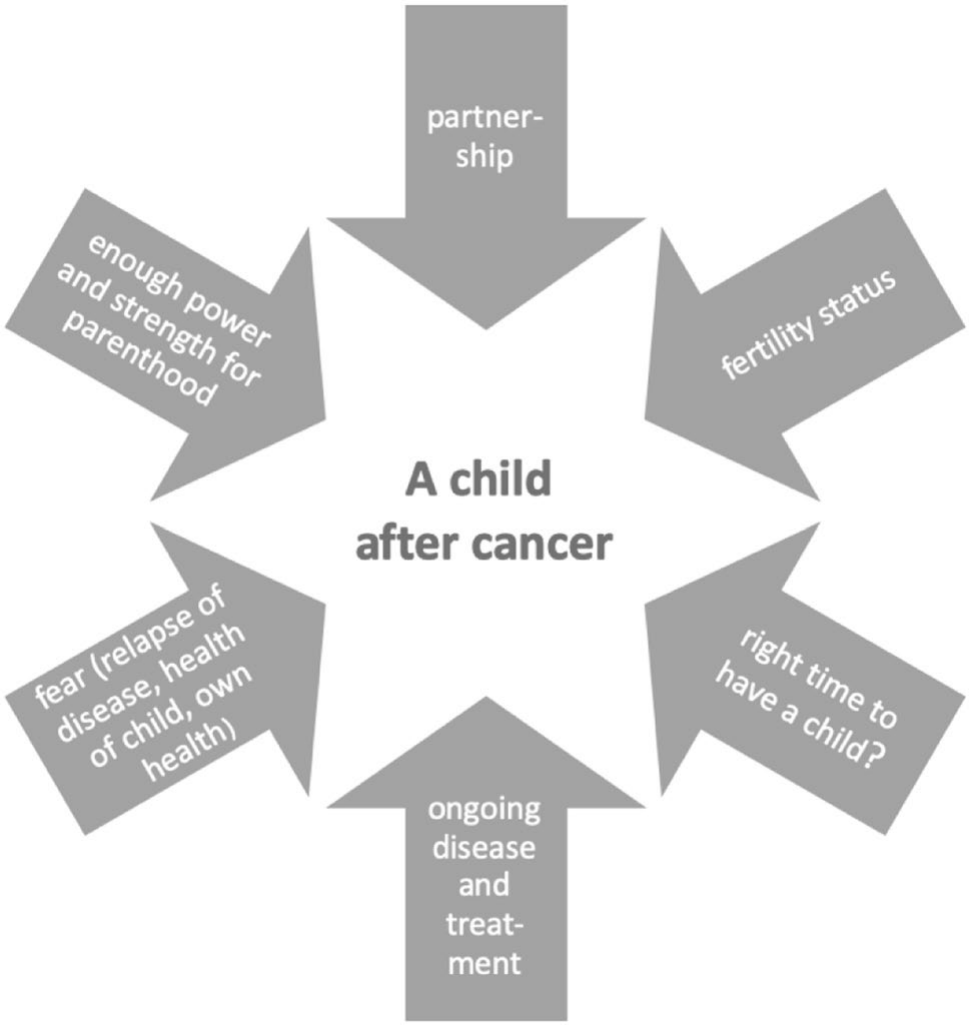
Factors influencing the desire to have a child after cancer

The findings of the questionnaire study and the second follow-up by telephone interview of women diagnosed with cancer show that family planning after cancer is a complex construct. Many factors interfere in this decision (Fig. 5). Fertility preservation techniques increase the chance for a woman to have her own child after cancer, but several other factors may outweigh the biological effects. Studies to evaluate the effect of fertility-preservation methods must, therefore, be interpreted with care.
